# Draft Genome Sequences of Corynebacterium diphtheriae Clinical Isolates from Colombia

**DOI:** 10.1128/MRA.00335-21

**Published:** 2021-07-22

**Authors:** Efraín Andrés Montilla-Escudero, Johan Fabian Bernal

**Affiliations:** a Grupo de Microbiología, Subdirección Laboratorio Nacional de Referencia, Dirección de Redes en Salud Pública, Instituto Nacional de Salud, Bogotá, Colombia; b Grupo de Investigación y Vigilancia de la Resistencia Antimicrobiana, Centro de Investigación Tibaitata Corporación Colombiana de Investigación Agropecuaria (AGROSAVIA), Bogotá, Colombia; University of Delaware

## Abstract

Since the implementation of the diphtheria-tetanus-pertussis (DTP) vaccine in Colombia, there has been a decrease in the reporting of cases. Here, we report two isolates of Corynebacterium diphtheriae bv. mitis isolated in the reference laboratory at Instituto Nacional de Salud from samples received from Norte de Santander and La Guajira; both areas are located on the northeast border of Colombia.

## ANNOUNCEMENT

Since the implementation of vaccination in Colombia, the number of reported cases of diphtheria in Colombia has decreased (1); however, in 2018, cases of diphtheria associated with migration were confirmed (2, 3). Diphtheria is a vaccine-preventable disease caused primarily by toxigenic strains of Corynebacterium diphtheriae, which manifests as a toxemic disease characterized by typical symptoms including pseudomembrane formation and severe obstruction in the respiratory tract, and diphtheria toxin can cause cardiac and neurological sequelae. In some parts of the world, it continues to be a public health problem, associated with low vaccination rates, migration, socioeconomic status, and other factors (4). The two strains reported here were isolated from male patients, one 14 years old from La Guajira (Distracción) and a fatal case of a 37-year-old reported in Norte de Santander (Cúcuta), both with unknown vaccination status.

Specimens were tested using the Coryne triplex real-time PCR assay developed by Williams et al. (5). This protocol was shared with us by Maria Tondella in 2018. The microorganism was cultured on tellurite cystine agar (6) for 48 h at 35°C in aerobiosis from pharyngeal swab samples. Species and biotype identification was confirmed with biochemical tests (7) and matrix-assisted laser desorption ionization–time of flight (MALDI-TOF) mass spectrometry (8). The two suspected diphtheria cases were confirmed using culture and Elek’s toxigenicity test (9).

Bacterial culture was grown in overnight aerobiosis to 37°C in blood agar. The genomic DNA was extracted using a PureLink genomic DNA minikit (Invitrogen, Carlsbad, CA, USA) with previous incubation at 37°C for 2 h with lysozyme (20 mg/ml). DNA libraries were prepared with the Nextera XT DNA sample preparation kit (Illumina, San Diego, CA, USA). The sequencing was performed using a whole-genome shotgun approach performed on an MiSeq system (Illumina) with V.2 251-cycle paired-end chemistry. Raw reads were trimmed and assembled using Trimmomatic v0.39 (github.com/timflutre/trimmomatic) and SPAdes v3.14.0 (github.com/ablab/spades) using default parameters. The sequences were automatically annotated with NCBI Prokaryotic Genome Annotation Pipeline (PGAP) v5 (10).

Strains GMR-D-25.18 and GMR-D-100.18 exhibited a total of 2,370 and 2,414 coding DNA sequences (CDS), respectively, and both genomes encoded 6 rRNAs, 1 repeat region, 54 tRNAs, and 1 transfer-messenger RNA (tmRNA). Diphtheria toxin, which inactivates translation elongation factor 2 (EF2), was localized for both genomes according to the annotation using Ariba (https://github.com/sanger-pathogens/ariba) and the VFDB database (11).

The multilocus sequence typing (MLST) analysis is a robust and simple tool developed in 2010 (12) that has the advantage of permitting comparability between isolates from the same species and transferability to the laboratories, and it includes the analysis of 7 genes, *atpA*, *dnaE*, *dnaK*, *fusA*, *leuA*, *odhA*, and *rpoB* (www.pubmlst.org/cdiphtheriae), due to the limited information on genomes available in the region for performing depth analysis with single nucleotide polymorphisms (SNPs) or a core genome MLST (cgMLST) scheme. We found that the GMR-D-25.18 isolate belongs to the sequence type 174 (ST174), which is the only profile reported to be circulating in Venezuela (3) and Brazil (13) associated with respiratory diphtheria. In this study, a new sequence type, ST677, is reported for the GMR-D-100.18 strain, which is close to ST174 with a variant in the *dnaE* allele gene (65 alleles with 99.78% identity) compared to the *dnaE* allele gene (2 alleles from ST174). This allele and profile were reported to the MLST database (https://pubmlst.org/bigsdb?page=profileInfo&db=pubmlst_cdiphtheriae_seqdef&scheme_id=1&profile_id=677) to be used in comparisons with other strains of C. diphtheriae that are analyzed or submitted in the PubMLST database, as reported by Strauss et al. (3). This profile may indicate the appearance of a new strain different from ST174 and ST697 that has been circulating on the border of Colombia and Venezuela since 2018 (3). However, the analysis of the complete genomes among the strains of the region is necessary to establish characteristics of the currently circulating bacterial populations in countries and to accomplish early identification of possible variants in vaccine targets.

The isolates from diphtheria surveillance were recovered under the framework of national laboratory surveillance led by the Microbiology Group from Dirección de Redes en Salud Pública-Instituto Nacional de Salud and belong to the response to events of interest in public health and sanitary control; therefore, informed consents were not generated, and the responsibility for the custody of isolates and all related information was handled anonymously. This study was conducted in adherence to the Declaration of Helsinki (https://www.wma.net/policies-post/wma-declaration-of-helsinki-ethical-principles-for-medical-research-involving-human-subjects/).

### Data availability.

The draft genome sequences of the two C. diphtheriae isolates have been deposited in the DDBJ/ENA/GenBank database under the accession numbers listed in Table 1. The raw sequencing data are available in the SRA under the numbers SRR13616374 and SRR13616375 and BioProject number PRJNA650156.

**TABLE 1:** Genome assembly details and statistics

Isolate	NCBI BioProject no.	NCBI BioSample no.	GenBank accession no.	No. of reads	Coverage (×)	*N*_50_ (bp)	Genome size (bp)	GC content (%)	No. of contigs
GMR-D-100.18	PRJNA650156	SAMN15693636	JAFIQB000000000	551,796	39.54	122,621	2,488,129	53.46	48
GMR-D-25.18	PRJNA650156	SAMN15694267	JAFIQC000000000	492,242	38.22	122,621	2,455,602	53.47	48
